# Genetic Variants Associated with Bronchial Asthma Specific to the Population of the Russian Federation

**DOI:** 10.32607/actanaturae.11853

**Published:** 2023

**Authors:** Y. N. Akhmerova, T. A. Shpakova, K. S. Grammatikati, S. I. Mitrofanov, P. G. Kazakova, A. A. Mkrtchian, P. U. Zemsky, M. N. Pilipenko, N. V. Feliz, L. V. Frolova, A. A. Frolovskaya, V. S. Yudin, A. A. Keskinov, S. A. Kraevoy, S. M. Yudin, V. I. Skvortsova

**Affiliations:** Federal State Budgetary Institution “Center for Strategic Planning and Management of Biomedical Health Risks” of the Federal Medical Biological Agency (Center for Strategic Planning of FMBA of Russia), Moscow, 119121 Russian Federation; Federal Medical Biological Agency (FMBA of Russia), Moscow, 123182 Russian Federation

**Keywords:** bronchial asthma, polymorphism, phenotype–genotype associations, genetic variants

## Abstract

Bronchial asthma (BA) is a disease that still lacks an exhaustive treatment protocol. In this regard, the global medical community pays special attention to the genetic prerequisites for the occurrence of this disease. Therefore, the search for the genetic polymorphisms underlying bronchial asthma has expanded considerably. As the present study progressed, a significant amount of scientific medical literature was analyzed and 167 genes reported to be associated with the development of bronchial asthma were identified. A group of participants (n = 7,303) who had voluntarily provided their biomaterial (venous blood) to be used in the research conducted by the Federal Medical Biological Agency of Russia was formed to subsequently perform a bioinformatic verification of known associations and search for new ones. This group of participants was divided into four cohorts, including two sex-distinct cohorts of individuals with a history of asthma and two sex-distinct cohorts of apparently healthy individuals. A search for polymorphisms was made in each cohort among the selected genes, and genetic variants were identified whose difference in occurrence in the different cohorts was statistically significant (significance level less than 0.0001). The study revealed 11 polymorphisms that affect the development of asthma: four genetic variants (rs869106717, rs1461555098, rs189649077, and rs1199362453), which are more common in men with bronchial asthma compared to apparently healthy men; five genetic variants (rs1923038536, rs181066119, rs143247175, rs140597386, and rs762042586), which are more common in women with bronchial asthma compared to apparently healthy women; and two genetic variants (rs1219244986 and rs2291651) that are rare in women with a history of asthma.

## INTRODUCTION

Bronchial asthma (BA) is a chronic, recurrent disease whose pathogenesis is associated with altered bronchial reactivity caused by both specific immunological and nonspecific mechanisms. The major (essential) clinical sign of BA involves choking episodes that result from bronchial spasm, mucus hypersecretion, and edema of the bronchial mucosa [1].

The WHO considers bronchial asthma to be among the most serious chronic, non-communicable diseases. Most deaths due to BA occur in low- and middle-income countries, which are characterized by insufficiently efficient diagnosis and treatment capabilities for the disease, as well as the healthcare system in general [2]. Up to 350 million people worldwide currently have BA [1], and this figure may increase to 450 million by 2025 [3].

According to official statistics, there are 1.3 million patients with BA in Russia. This means that the prevalence of this disease in Russia is less than 1%, while the proportion of people with BA is less than 0.4% of all patients with asthma worldwide. Meanwhile, the European Respiratory Society has estimated the incidence of BA in a number of European countries at 5% among adults and more than 7% among children. A trend towards growing rates of disability and death due to BA is observed in many countries. Thus, the rate of BA deaths in Great Britain has increased sevenfold over the past 20 years, and two- to threefold, in North America. More than 5,000 people die due to BA in the U.S. each year.

BA develops due to a number of factors, including the intensity of allergen exposure, habitat destruction, overactive immune response, and individual genetic features [3]. It has been demonstrated that there is a 25% risk that a child whose parent suffers from bronchial asthma also develop this disease. If both parents have asthma, the risk increases to 50% [4]. Furthermore, it has been proved that there exists an association between the increasing incidence rate of BA and aggravated soil, air, and water contamination [5].

In 2018, the direct expenses of the Russian healthcare system for BA treatment amounted to ~ 8.5 billion rubles; two-thirds of this amount was spent on hospital stays. Moreover, substantial funds are needed to cover temporary disability leaves and disability payments [6]. Early diagnosis and prevention of BA will make it possible both to reduce these costs and the prevalence of bronchial asthma in Russia.

## EXPERIMENTAL

**Building the cohorts**

The manifestation and course of BA is significantly different in men than it is in women, which is largely due to the different contributions of reproductive hormones to the pathogenesis of BA [7]. Therefore, our study participants were divided into cohorts according to such factors as history of BA and sex.

The 7,303 study participants were allotted to four cohorts:

1A – women with a confirmed diagnosis of BA (mean age, 52 ± 10 years), n = 218;

2A – men with a confirmed diagnosis of BA (mean age, 41 ± 12 years), n = 70;

3H – apparently healthy women without a history of BA or other diagnoses with a similar clinical presentation (mean age, 52 ± 8 years), n = 4,015;

4H – apparently healthy men without a history of BA or other diagnoses with a similar clinical presentation (mean age, 44 ± 6 years), n = 3,000.

An inclusion criterion for groups 1A and 2A was a history of bronchial asthma in the anamnesis. Groups 3H and 4H contained visibly healthy men and women; the exclusion criteria for these cohorts were medical records indicating that a patient had a history of diseases whose symptoms were similar to manifestations of BA, such as acute bronchitis, pulmonary emphysema, allergic rhinitis, gastroesophageal reflux, tracheoesophageal fistula, congenital heart disease, tracheomalacia and bronchomalacia, cystic fibrosis, primary ciliary dyskinesia, bronchiectasis of other etiologies, tuberculosis, lung cancer, a vascular ring anomaly, sarcoidosis, intrathoracic lymphadenopathy, bronchopulmonary dysplasia, allergic bronchopulmonary aspergillosis, systemic anaphylaxis, primary immunodeficiency, vocal cord dysfunction, psychogenic cough, and affective respiratory paroxysms [8].

**Biomaterial sampling and personal data of study participants**

Samples from collections previously created by the Center for Strategic Planning of FMBA of Russia were used in this study. In all cases, the data were collected in full compliance with the procedural requirements: the following respective data were included and verified for each donor: sex, age, region of residence, nationality, past medical history, informed consent (signed by the donor) for biomaterial sampling, handling, transportation, storage and personal data use; proper procedures for sample (venous blood) collection, handling, transportation, and storing was ensured, per the State Standard GOST R53079.4-2008.

All the specimens selected for the final study sample were checked to make sure that the donor’s ID code and the information deciphered in that code were unique. Furthermore, it was guaranteed that the biomaterial had no signs of hemolysis or lipemia. The samples were transported under constant temperature control.

**Creating a candidate gene list**

Over 150 genes associated with the development of BA have been reported thus far. The following tentative gene groups are of special interest:

the atopy-related genes. These genes include IL4, IL5, IL13, IL4RA, CHI3L1, RAD50, etc. and are responsible for the blood level of total and specific IgE, as well as the development of allergic responses;

the genes related to bronchial hyperreactivity, including ADRB2, TNF, IL5, IL9, NOS1, NPSR1, TAC1, TACR2, TACR1, TACR3, ADAM33, ACE, etc., being responsible for bronchial hyperresponsiveness, which is tightly related to the blood IgE level and inflammation;

the inflammation-related genes such as TNF, IL4, IL5, IL13, ORMDL3, SCGB3A2, CCL11, IRAK3, CSF2, ALOX5, CYSLTR1, CYSLTR2, LTC4S, STAT3, STAT6, etc., being responsible for the level of inflammatory mediators by their role in regulating the immune response and behavior of inflammatory cells in body fluids [8].

A list comprising 167 candidate genes was created according to 107 references to search for phenotype– genotype associations. The list of these genes, with a brief description of the functions of the proteins encoded by them, is provided in Discussion.

**DNA isolation, construction of genomic libraries, and sequencing**

DNA was isolated from whole blood samples using a MagAttract HMW DNA Kit (Qiagen, Germany). The DNA isolation protocol was automated on the Tecan Freedom EVO workstation (Tecan, Switzerland). The concentration and purity of the isolated DNA were measured on a Tecan Infinite® F Nano Plus microplate reader (Tecan, Switzerland).

The genomic libraries for sequencing were prepared using a Nextera DNA Flex kit (Illumina, USA), in accordance with the manufacturer’s recommendations. Each sample in the flow cell was labelled using indexes from the IDT-ILMN Nextera DNA UD kit (Illumina, USA).

The concentrations of the genomic libraries were measured using a Tecan Infinite® F Nano Plus spectrophotometer. The size of the genomic libraries was determined on an Agilent TapeStation 4200 system using an Agilent DNA 1000 kit (Agilent, USA). The library pools consisting of 24 samples were combined on a Tecan Freedom EVO robotic platform.

Genome-wide sequencing was performed on a NovaSeq 6000 sequencing system and a S4 reagent kit (300 cycles) (Illumina, USA) for paired-end reads 2 × 150 bp.

**Bioinformatic analysis of the whole-genome sequencing data**

Demultiplication was performed at the first stage of the analysis of the primary sequencing data. During this procedure, the initial reads of the NovaSeq 6000 sequencing system was converted from the BCL format to the FASTQ format using the Illumina bcl2fastq conversion software v2.20 [9]. The Illumina Sequencing Analysis Viewer software v2.4.7 was employed to control the overall sequencing quality of the entire cell [10]. The quality of individual reads was controlled using the FastQC v0.11.9 bioinformatic tool [11].

The final sample contained blood specimens that had undergone quality control for such parameters as homogeneity of the nucleotide distribution in the reads and GC composition.

Read alignment against a reference genome was performed at the second stage of the bioinformatic analysis using the DRAGEN platform [12]. The GRCh38.d1.vd1 sequence was used as the reference genome. Blood samples with average coverage over genome < ×30 were excluded from the study.

The CrosscheckFingerprints software (Picard) [13] was used to check whether the sample contained any duplicates. All duplicate specimens were excluded from the study.

**Search for short genetic variations**

The Strelka software was used to process VCF files and search for short genetic variations (SNPs, indels up to 50 bp long) [14].

Finally, 380,564 short genetic variations were detected in 167 candidate genes (7,303 samples); 253,628 of those were found more than once.

The procedure for searching for genetic variations whose frequency was statistically significantly different in different cohorts was employed to identify the polymorphisms associated with BA. The Fisher’s exact test was used to determine the significance level of the differences.

The case with identical occurrence of the "zero" variant in all four cohorts was assumed to be the null hypothesis. The significance level at which the null hypothesis was rejected was set equal to 10-4. The calculations were performed using the R programming language.

## RESULTS

**Comparison of male cohorts**

**Table 1:** SNPs associated with bronchial asthma in the cohorts of men

Chromosome	Polymorphism ID number"	Gene"	Frequency		p
			cohort 2A, %	cohort 4H, %	
chr3	rs869106717 (del)	FOXP1	5.71	0.17	1.1 × 10^-5^
chr4	rs1461555098 (del)	TACR3	12.86	2.07	2.9 × 10^-5^
chr7	rs189649077 (G>T)	EGFR	4.29	0.03	2.4 × 10^-5^
chr19	rs1199362453 (T>C)	ZNF257	4.29	0.00	4.5 × 10^-5^

Our analysis revealed four genetic variants in the introns of the TACR3, ZNF257, FOXP1, and EGFR genes; their frequencies differ statistically significantly (the p value being no higher than the significance level of 10^-4^) in the cohorts of men with a verified diagnosis of BA and in the cohort of apparently healthy men. These genetic variants are found significantly more frequently in cohort 2A (more than fivefold) (Table 1) than in cohort 4H.

The TACR3 gene that encodes tachykinin receptors and has an indirect effect on the bronchial tone [15, 16] was found to carry the rs1461555098 deletion (chr4:g.103629850_103629861del). According to our calculations, the relative risk of developing BA in individuals carrying this deletion stands at 6.9, while this parameter is normally equal to 1.0. The deletion rs1461555098 (chr4:g.103629850_103629861del) was detected in cohort 2A twice as frequently as in cohort 4H.

The ZNF257 gene encoding the transcription factor (a zinc finger motif-containing protein) was found to carry the genetic variant rs1199362453 (chr19:g.22076863T>C), which was encountered three times in cohort 2A but was absent in cohort 4H.

The FOXP1 gene encoding the transcription factor and expressed in the proximal airway epithelium [18] was found to carry the genetic variant rs869106717 (chr3:g.71465326del), which was encountered in cohort 2A 33.6 times more frequently than in cohort 4H. The relative risk of developing BA in individuals carrying this mutation is 36.0.

In the EGFR gene that encodes the transmembrane receptor binding extracellular ligands belonging to the epidermal growth factor group [19], the frequency of the genetic variant rs189649077 (chr7:g.55168296G>T) in cohort 2A was 143-fold higher compared to that in cohort 4H. The relative risk of developing BA in individuals carrying this mutation is 34.3.

**Comparison of female cohorts**

It was demonstrated that in the cohort of women with a confirmed diagnosis of BA, five genetic variants were six times more frequent compared to the cohort of apparently healthy women (the p value is no higher than the significance level of 10-4). These genetic variants resided in the CYSLTR1, IL5RA, NRG1, HDC, and DPP10 genes (Table 2).

**Table 2:** SNPs associated with bronchial asthma in the cohorts of women

Chromosome	Polymorphism ID number	Gene	Frequency		p
			cohort 1A, %	cohort 3H, %	
chrX	rs1923038536 (G>A)	CYSLTR1	2.29	0.05	6.7 × 10^-6^
chr1	rs1219244986 (T>C)	MUC1	1.83	9.46	1.5 × 10^-5^
chr3	rs2291651 (G>C)	MUC4	83.03	91.88	5.0 × 10^-5^
chr3	rs181066119 (A>G)	IL5RA	1.83	0.05	9.5 × 10^-5^
chr8	rs143247175 (T>A)	NRG1	1.83	0.05	9.5 × 10^-5^
chr15	rs140597386 (dup)	HDC	4.13	0.67	6.0 × 10^-5^
chr2	rs762042586 (del)	DPP10	1.83	0.05	9.5 × 10^-5^

The CYSLTR1 gene that encodes the protein affecting the secretion of inflammatory mediators (leukotrienes) [16, 20] was found to carry the rs1923038536 (chrX:g.78306516G>A) variant, which was detected in cohort 1A 45.8 times more frequently compared to cohort 3H. The relative risk of developing BA in individuals carrying this mutation is 14.2.

T h e g e n e t i c va r i a n t r s 1 8 1 0 6 6 1 1 9 (chr3:g.3102851A>G) was found in the IL5RA gene encoding the subunit of the heteromeric receptor of interleukin 5, a cytokine that plays a crucial role in eosinophil differentiation [21]; this variant occurred in cohort 1A 36.6 times more frequently than in cohort 3H. The relative risk of developing asthma in individuals carrying this mutation is 13.2.

T h e g e n e t i c va r i a n t r s 1 4 3 2 4 7 1 7 5 (chr8:g.32692193T>A) was found in the NRG1 gene encoding mucin production by airway goblet cells [22]; like the previous genetic variant, it was found in cohort 1A 36.6 times more frequently than in cohort 3H. The relative risk of developing BA in individuals carrying this mutation is also 13.2.

The HDC gene codes for the enzyme catalyzing histamine synthesis from L-histidine [23]. The genetic variant rs140597386 (chr15:g.50261726dup) was identified for this gene; it occurred in cohort 1A 6.2 times more frequently than in cohort 3H. The relative risk of developing BA in individuals carrying this mutation was 5.0.

T h e g e n e t i c va r i a n t r s 7 6 2 0 4 2 5 8 6 (chr2:g.115490670del) was identified in the DPP10 gene encoding membrane-anchored serine protease [16], which was found in cohort 1A 36.6 times more frequently than in cohort 3H. The relative risk of developing BA in individuals carrying this mutation is 13.2.

T h e g e n e t i c v a r i a n t s r s 2 2 9 1 6 5 1 ( c h r 3 : g . 1 9 5 7 5 1 1 4 1 G > C ) a n d r s 1 2 1 9 2 4 4 9 8 6 (chr1:g.155189991T>C) of the MUC1 and MUC4 genes occurred in women with asthma much less frequently than in apparently healthy women. This means that the identified genetic variants can be considered protective in individuals with BA [24]. The MUC1 and MUC4 genes encode mucins. The MUC1 gene is responsible for the anti-inflammatory effect in patients with bronchial and lung diseases. MUC4 exhibits a mediated effect on the proliferation of airway epithelial cells [25]. The genetic variant rs2291651 (chr3:g.195751141G>C) of the MUC4 gene in cohort 1A occurred somewhat more rarely than in cohort 3H. The relative risk of developing BA in individuals carrying this mutation is 0.5, while normally this parameter is 1.0. This indicates that the risk of developing bronchial asthma is down twofold in women carrying the genetic variant rs2291651. The relative risk of developing BA in females carrying the genetic variant rs1219244986 (chr1:g.155189991T>C) is 0.2 (i.e., lower than 1), corresponding to the fivefold reduction in the risk of developing the disease.

However, allowance should be made for the fact that the identified variants in the MUC1 and MUC4 genes reside in GC-rich regions. This region negatively affects sequencing quality and, therefore, the quality of the genetic variant detection.

## DISCUSSION

We have analyzed the candidate genes potentially associated with BA. Eleven polymorphisms whose frequency differs significantly in individuals diagnosed with BA and those not diagnosed with the condition have been identified. Nine of the identified genetic variants increase the risk of developing BA, while two variants reduce it. These nine variants increase the risk of developing BA at least fivefold. The identified variants are specific to the population of Russia.

Arathimos et al. [26] reported that up to 45% of females with bronchial asthma experience an aggravation of their condition before their menstrual period. In 2020, the polymorphism rs2291651 in the MUC4 gene was described to be an accompanying sign of endometriosis in South Korean women [27]. The relationship between the single nucleotide polymorphisms in the MUC1 and MUC4 genes and endometriosis risk was analyzed in this study. Screening identified eight genetic variants of MUC4, including rs2291651, whose presence correlated with the development of endometriosis. Women of childbearing age using oral contraceptive pills tended to experience milder asthma attacks [28]. A number of studies [29, 30] also demonstrated that variations in the estradiol and progesterone levels during the menstrual cycle affected the severity of bronchial asthma symptoms. That means that when studying the genetic predisposition to a severe BA course in women, one should pay particular attention to the genes associated with female sex hormones.

In this study, we have analyzed 167 candidate genes associated with bronchial asthma. These genes include HNMT, MS4A2, HRH1, HRH2, HRH3, HRH4, AOC1, and HDC, which code for the histamine receptors that are involved in the regulation of histamine release [16, 23, 31, 32, 33].

The HDC gene encodes the enzyme histidine decarboxylase catalyzing histamine formation from L-histidine; the HDC mRNA level is elevated in patients with asthma [23];

the IL3, IL4, IL4R, IL5, IL9, IL13, IL17, IL21R, IL18, IL18R1, IL2RB, IL1RL1, IL5RA, IL33, SCGB3A2, TNF, CCL11, IRAK3, CSF2, and TSLP genes encode the cytokines involved in inflammation. Thus, IL5 stimulates eosinophil release into the bloodstream, while IL5RA regulates their activity. Stimulation of the airways with allergens increases the local IL5 concentration, which correlates with the severity of airway eosinophilia, while IL4RA codes for the α chain of the IL4 receptor, which can bind IL4 and IL13 to regulate the IgE production [4, 16, 21, 23, 34, 35, 36, 37, 38, 39, 40];

the IL17F gene encodes the pro-inflammatory cytokine involved in pathophysiological manifestations of asthma. In vivo and in vitro studies have shown that IL17F is involved in the pathogenesis of allergic airway inflammation [41];

the ADRB2 gene encodes the β2-adrenoceptors that play a crucial role in airway contractility. β2-adrenoceptors act as a target for β2-agonists exhibiting a marked bronchodilator and bronchoprotective activity, which is important for assessing the effectiveness of BA therapy [16];

the PLA2G7 gene encodes the platelet-activating factor acetylhydrolase. This enzyme catalyzes the cleavage of PAF by hydrolyzing the acetyl group down to biologically inactive products [31];

the ALOX5, CYSLTR1, CYSLTR2, and LTC4S genes encode the proteins that affect the production of inflammatory mediators (leukotrienes), contributing to various allergic and hypersensitivity reactions. It has been shown that altered expression of some of these genes may cause bronchoconstriction of the airways and hyperresponsiveness to bronchoconstricting agents such as histamine, increased vascular permeability, edema, eosinophilia and neutrophilia, smooth muscle cell proliferation, collagen deposition and fibrosis in different tissue areas, mucin secretion by goblet cells, metaplasia of goblet cells, and hypertrophic changes in the respiratory epithelium [16, 20, 42];

the PTGER2 and PTGDR genes encode prostaglandin receptors and are involved in the pathogenesis of BA [16, 43];

the TBX21 and TBX5 genes encode transcriptional activators; their expression is downregulated in airway-resident T cells in asthma patients [16, 44];

the STAT6 gene encodes the STAT family transcription factor; expression of this gene is significantly upregulated in patients with severe BA [16];

the STAT3 gene encodes the STAT family transcription factor mediating the cellular responses to interleukins and regulates the inflammatory response [45, 46];

the STAT4 gene encodes the STAT family transcription factor; expression of this gene is downregulated in patients with BA [47];

the NPSR1 gene encodes the neuropeptide S receptor; the upregulated expression of this gene in airway epithelium leads to the activation of matrix metalloproteinases, which are involved in the pathogenesis of BA [16, 48];

the TAC1, TACR2, TACR1, and TACR3 genes encode receptors for tachykinins, which are found in sensory nerve endings, are activated by inflammatory mediators (histamine, platelet-activating factor, and leukotrienes), and add the axon reflex mechanism to the pathogenesis of asthma, thus leading to aggravation and spread of the initial inflammation. Tachykinins affect the bronchial tone and vascular permeability [16];

the CHI3L1 gene encodes the glycoprotein belonging to the glycoside hydrolase family and contributes to the development of the Th2-type inflammatory response [16, 49];

the DENND1B gene encodes the protein interacting with tumor necrosis factor and plays a crucial role in suppressing T-cell receptors on Th2 cells [50, 51];

the ADAM33 gene codes for metalloprotease. ADAM33 is expressed by various types of airway cells. ADAM33 expression is elevated in patients with BA; the impaired function of this metalloprotease can be associated with bronchial hyperresponsiveness and airway wall remodeling, thus contributing to early manifestation of bronchial asthma [52];

the ORMDL1, ORMDL2, and ORMDL3 genes encode ORM-like proteins, the key regulators of serine palmitoyltransferase, which catalyzes the first step of sphingolipid biosynthesis. Sphingolipids play an important role in signal transduction in response to stress and affect the mechanical properties of cell membranes. Dysregulation of sphingolipid biosynthesis is associated with several diseases, including allergies, inflammation, and asthma [53, 54];

the VIP gene encodes the vasoactive intestinal peptide responsible for the relaxation of smooth muscles [55];

the genes belonging to the NOS family encode nitric oxide synthases. Mutations in the NOS1 gene reduce the nitric oxide concentration in non-eosinophilic phenotype patients, which is a marker of bronchial asthma, and cause bronchial hyperresponsiveness [56, 57, 58];

the ACE gene encodes angiotensin, which converts angiotensin I to the vasoactive angiotensin II, and is involved in the pathogenesis of BA as it causes proliferation and increases smooth muscle contractility, thus leading to lung obstruction [59];

protein RAD50 encoded by the RAD50 gene is involved in double-strand DNA break repair. It was shown in transgenic mice that the fragment of the 3’-terminus of this gene is the Th2 locus control region (LCR), which regulates cytokine gene expression [60];

the PTAFR gene encodes the receptor for the platelet-activating factor, a chemotactic phospholipid mediator exhibiting strong inflammatory, contractile, and hypotensive activities with respect to smooth muscles. The PAF receptor is involved in various pathological processes, such as allergies, asthma, septic shock, arterial thrombosis, and inflammation [16];

the OPN3 gene encodes the G-protein-coupled receptor. Upregulated OPN3 expression was detected in bronchial epithelium and immune cells. Mutations in the OPN3 gene increase the risk of bronchial asthma [20, 61];

the GSDMB gene encodes the protein whose overexpression in bronchial epithelial cells increases expression of the genes that are crucial for both airway remodeling and airway hyperresponsiveness [16, 62];

the PKN2 gene encodes serine/threonine-specific protein kinase and regulates apical junction formation in human bronchial epithelium [63];

the PTK2 gene codes for tyrosine protein kinase and plays a crucial role in airway hyperresponsiveness and airway remodeling [63];

the ALPP gene encodes the placental alkaline phosphatase catalyzing the hydrolysis of phosphoric acid monoesters; the expression level of this gene is associated with childhood asthma [63];

the PTEN gene encodes phosphatidylinositol-3,4,5- triphosphate-3-phosphatase [20]. A low PTEN expression level is considered to be among the independent factors of BA development [64];

the PRMT1 gene encodes an important epigenetic regulator, protein arginine methyltransferase-1, which contributes to inflammation and airway remodeling in patients with BA [65];

the HSPD1 gene encodes the heat shock protein that can modulate the immune and inflammatory responses, be involved in pathogenesis, and/or be a risk factor or a prognostic marker for several diseases, including BA [66];

the TLR2 and TLR4 genes encode proteins belonging to the Toll-like receptor family, which are essential for pathogen recognition and activation of the innate immune system. Some polymorphisms in these genes are associated with the risk of developing BA [67];

the ZNF208, ZNF257, ZNF676, ZNF729, ZNF98, ZNF492, ZNF99, ZNF723, ZNF728, ZNF730, and ZNF91 genes encode zinc finger proteins residing within the region of the transcription factor cluster area and are associated with the pathogenesis of BA [17];

the B4GALT1 gene encodes beta-1,4-galactosyltransferase and is associated with the atopic phenotypes and inflammatory conditions [68];

the IGFBP3 gene codes for a protein binding insulin-like growth factor and inhibits the specific physiological effects of asthma in an IGF-independent manner [69];

genes belonging to the MUC family encode mucins. MUC7 codes for salivary mucin; the frequency of the MUC7 allele with five tandem repeats is significantly reduced in patients with asthma [20, 70]. During the late stages of bacterial infection, MUC1 exhibits an anti-inflammatory activity in the airways, which is initiated and mediated by inhibition of Toll-like receptor signaling [24]. Mucin MUC4 was identified as a ligand-activating receptor tyrosine kinase, which modulates the proliferation of airway epithelial cells in patients with asthma [25]; MUC19 is mainly expressed in the cells of submucous glands in the trachea and salivary glands; in patients with allergic rhinitis and chronic otitis media, this gene is expressed in the epithelium. MUC5AC is expressed in the goblet cells of tracheal and bronchial epithelium. MUC5B is also expressed in the submucosal epithelium and ducts and, to a lesser extent, in the goblet cells of both tracheal and bronchiolar epithelium. Many individuals with a confirmed diagnosis of bronchial asthma have elevated levels of MUC5AC mRNA but reduced levels of MUC5B mRNA [71];

the NRG1 gene encodes the protein-inducing production of mucins MUC5AC and MUC5B by human airway goblet cells, so its inhibition can be regarded as a novel therapeutic approach to reducing mucus hypersecretion in patients with respiratory diseases [22];

the DACT1, DACT2, and DACT3 genes code for the proteins involved in the pathogenesis of BA. The tissue levels of DACT1, DACT2, and DACT3 mRNA are significantly elevated in asthma patients [72];

the CYP genes encode the cytochrome proteins involved in the metabolism of many drugs, including nonsteroidal anti-inflammatory drugs, oral anticoagulants and angiotensin receptor blockers, as well as in the synthesis of cholesterol, steroids, and other lipids [20, 70, 73, 74, 75, 76, 77];

the CHML gene codes for Rab geranylgeranyltransferase regulating the intracellular transport of membrane structures. Polymorphisms in this gene are associated with the development of BA [61];

the GSTT2 and GSTP1 genes encode glutathione S-transferase theta 2 and glutathione S-transferase P; polymorphisms in these genes may be risk factors for BA [78];

the NAT2 gene codes for N-acetyltransferase 2; polymorphisms in this gene are associated with the development of atopic asthma [79];

the PYHIN1 gene encodes the interferon-inducible HIN-200 protein, which is involved in the production of proinflammatory cytokines in airway epithelial cells [80];

the SMAD3 gene promoter is significantly hypermethylated in patients with BA [81];

the PGAP3 gene encodes a glycosylphosphatidylinositol-specific phospholipase predominantly residing in the Golgi apparatus. The PGAP3 and ORMDL3 proteins can contribute to the development of BA [82];

the ERBB2 gene encodes the epidermal growth factor receptor tyrosine kinase. The ERBB2 expression level in freshly isolated airway epithelium in asthma patients is lower than that in healthy individuals [83];

the COL15A1 gene coding for the alpha chain of collagen type XV, a member of the FACIT collagen family [16], is involved in the metabolism of the drugs used to treat lung diseases [84];

the FOXP1 gene encodes the transcription factor belonging to the FOXO family, which is expressed in the proximal airway epithelium of the lungs; downregulated expression of FOXP1 inhibits early differentiation of secretory cells [18];

the ACOT7 gene encodes a protein belonging to the acyl-coenzyme family; an epigenome-wide association study revealed an association between the degree of methylation and the development of bronchial asthma [85];

the MTHFR gene codes for the methyltetrahydrofolate reductase. Polymorphisms in the MTHFR gene are associated with predisposition to bronchial asthma and glucocorticoid responsiveness in humans [86];

the DICER1 gene encodes RNA helicase involved in cytokine production and signal transduction in patients with BA [87];

the SERPINC1 gene encodes antithrombin III, which inhibits clotting factors; variations in its level may induce thrombosis or pulmonary embolism [88];

the SYNM gene codes for an intermediate filament; there is a hypothesis that the degree of methylation of this gene is associated with the development of BA [89];

the GATA3 gene encodes a transcription factor belonging to the GATA family. The GATA3 expression level in the airways is significantly increased in patients with asthma. The increased GATA3 expression level correlates with changes in IL5 expression and the development of bronchial hyperresponsiveness [90];

the FOXP3 gene encodes an activating transcription factor; the expression level of this gene is downregulated in asthma patients [91];

the CCDC80, DAPK3, LOXL1, PROC, FUCA2, SP100, and ITCH genes encode proteins associated with antigen presentation to T lymphocytes. The degree of methylation of these genes was found to be increased in asthma patients [76];

the VDR gene codes for the vitamin D3 receptor. Genetic variants of the VDR gene are often found in children with BA; their presence inversely correlates with asthma severity [92];

the DPP10 gene encodes a membrane protein belonging to the serine protease family. Mutations in this gene increase the risk of BA [16, 93];

the genetic variants of the PHF11, SPP1, and PLAUR genes are associated with elevated IgE levels [94];

the SLC22A5 gene encodes an organic cation transporter; its expression level in the bronchial epithelium is reduced in asthma patients [95];

the EPHX1 gene codes for microsomal epoxide hydrolase. A high EPHX1 expression level is associated with an increased risk of developing BA at any time in one’s life [96];

the CTLA4 gene encodes one of the proteins from the immunoglobulin superfamily. According to the meta-analysis data, some polymorphisms in this gene are risk factors for developing BA [16, 97];

the MMP9 gene encodes a matrix metalloprotease involved in local proteolysis of the extracellular matrix, leukocyte migration, and airway remodeling [98];

the SOCS5 gene codes for a protein belonging to the family of cytokine signaling inhibitors. The single-nucleotide polymorphisms identified in this gene are associated with the development of BA [99];

the polymorphisms in the FCER2 gene encoding CD23 are associated with atopy, higher risk of exacerbation in patients with asthma, and a high serum IgE level [100];

the VEGFA gene encodes the heparin binding protein, one of the PDGF/VEGF growth factors. An elevated expression level of this gene is detected in patients with BA [101];

the ASB3 gene codes for the protein involved in smooth muscle cell proliferation and muscle cell development. A genome-wide association study revealed an association between polymorphisms in this gene and the development of BA [102];

the CRISPLD2 gene encodes the secretory protein LCCL, which increases glucocorticoid sensitivity and regulates the immune response [103];

according to a genome-wide association study, polymorphisms in the APOBEC3B, APOBEC3C, and EDDM3B genes are associated with asthma exacerbations [104];

a whole-genome association study revealed an association between polymorphisms in the BBS9 gene and the effectiveness of asthma treatment in children [105];

the PRKG1 gene encodes cGMP-dependent protein kinase, a key mediator of the nitric oxide (NO)/cGMP signaling pathway, and contributes to smooth muscle relaxation [16];

the DNAH5 gene codes for the dynein protein. The DNAH5 expression level in the bronchial epithelium is reduced in asthma patients compared to that in the control group [106];

the JAK1 and JAK2 genes encode the tyrosine kinases involved in the inflammatory cytokine signaling pathways associated with a higher frequency of asthma exacerbation and increased susceptibility to allergic sensitization and environmental antigens [107, 108];

the CHRNA1 and CHRNA3 genes code for nicotinic acetylcholine receptors. Polymorphisms in these genes are considered to be genetic risk factors for bronchial obstruction [109];

the TGF-*β *gene encodes a secreted ligand belonging to the TGF-β protein superfamily. TGF-β isoforms play a role in the regulation of airway inflammation and remodeling [110];

variants in the HHIP gene are associated with chronic obstructive pulmonary disease [111];

the SOD3 gene encodes superoxide dismutase. The SOD3 expression level is elevated in patients with BA, and some genetic variants of this gene affect the distribution of extracellular superoxide dismutase in the lungs and reduce the risk of manifesting BA symptoms [112];

the EGFR gene encodes a transmembrane receptor binding extracellular ligands belonging to the epidermal growth factor group. Biopsy specimens from asthma patients often contain regions of epithelial damage that are immunostained with EGFR; an elevated EGFR expression level is also observed in the morphologically intact epithelium of asthma patients [19];

the SLC11A1 gene codes for the divalent metal transporter protein carrying iron and manganese. A number of studies have revealed an association between polymorphisms in this gene and the development of lung diseases [113]; and

the ZPBP2 gene encodes a protein expressed in the bronchial glandular epithelium. The degrees of methylation of this gene are different in healthy individuals than they are in patients with BA [114].

## CONCLUSIONS

The genetic variants of a number of genes identified in this study, which increase and reduce the relative risk of developing BA, may facilitate early diagnosis of bronchial asthma and accurate diagnosis-making in case of ambiguity. In the long run, analysis of the samples collected from residents of different regions will help assess the geographic distribution of the risk-editing genetic variants and not only perform mapping of BA prevalence, but also adequately allocate financial and material resources, as well as qualified medical staff, across regions. Timely, including prenatal, detection of individuals genetically predisposed to BA and accurate diagnosis-making will improve the quality of medical care, reduce the rates of disability and death due to bronchopulmonary events, and decrease the direct and indirect cost of combatting bronchial asthma.

## Abbreviations

BA: bronchial asthma
WHO: World Health Organization
AHR: airway hyperreactivity
GCs: glucocorticoids
IGCs: inhaled glucocorticoids
FEV: forced expiratory volume
bp: base pair
SNP: single-nucleotide polymorphism
PAF: platelet-activating factor

## References

[1] (2021). Interregional public organization Russian Respiratory Society, All-Russian public organization Association of Allergists and Clinical Immunologists, All-Russian public organization Union of Pediatricians of Russia, Scientific Council of the Ministry of Health of the Russian Federation. Clinical guidelines. Bronchial asthma. M., 2021. P. 7..

[2] Vos T., Lim S.S., Abbafati C., Abbas K.M., Abbasi M., Abbasifard M., Abbasi-Kangevari M., Abbastabar H., Abd-Allah F., Abdelalim A. (2020). Lancet..

[3] Zaikina M.V. (2017). Bronchial asthma in young men: early changes in the functional state of the cardiorespiratory system, Perm State Medical University named after Academician E.A. Wagner of the Ministry of Health of the Russian Federation, 2017. 153 p..

[4] Asanov A.Yu., Namazova L.S., Pinelis V.G., Zhurkova N.V., Voznesenskaya N.I. (2008). Pediatric Pharmac..

[5] The European Community Respiratory Health Survey II. (2002). Eur. Resp. J. Eur. Resp. Soc..

[6] Belevsky A.S., Zaitsev A.A. (2018). Med. advice..

[7] Yung J.A., Fuseini H., Newcomb D.C. (2018). Ann. Allergy Asthma Immunol..

[8] Chuchalin A.G., Abelevich M.M., Arkhipov V.V., Astafieva N.G., Asherova I.K., Balabolkin I.I., Baleva L.S., Baskakova A.E., Blokhin B.M., Bogorad A.E. (2012). National program Bronchial asthma in children. Treatment strategy and prevention. M.,.

[9] Holtgrewe M., Messerschmidt C., Nieminen M., Beule D. (2020). Bioinformatics..

[10] Wright M.N., Gola D., Ziegler A. (2017). Statistical Human Genetics..

[11] de Sena) Brandine G., Smith A.D. (2021). F1000Res..

[12] Miller N.A., Farrow E.G., Gibson M., Willig L.K., Twist G., Yoo B., Marrs T., Corder S., Krivohlavek L., Walter A. (2015). Genome Medicine..

[13] Najafov J., Najafov A. (2017). Nature.

[14] Saunders C.T., Wong W.S.W., Swamy S., Becq J., Murray L.J., Cheetham R.K. (2012). Bioinformatics..

[15] Ramalho R., Soares R., Couto N., Moreira A. (2011). BMC Pulm Med..

[16] Stelzer G., Rosen N., Plaschkes I., Zimmerman S., Twik M., Fishilevich S., Stein T.I., Nudel R., Lieder I., Mazor Y. (2016). Curr. Protoc. Bioinformatics..

[17] Karunas A.S. (2013). Identification of genes of predisposition to bronchial asthma in various ethnic groups of the Volga-Ural region of Russia. Russian Fund for Basic Research. 2013. Grant № 13-04-01397..

[18] Li S., Wang Y., Zhang Y., Lu M.M., DeMayo F.J., Dekker J.D., Tucker P.W., Morrisey E.E. (2012). Development..

[19] Puddicombe S.M., Polosa R., Richter A., Krishna M.T., Howarth P.H., Holgate S.T., Davies D.E. (2000). FASEB J..

[20] (2021). Nucleic Acids Research.

[21] Cheong H.S., Kim L.H., Park B.L., Choi Y.H., Park H.S., Hong S.J., Choi B.W., Park C.S., Shin H.D. (2005). J. Hum. Genet..

[22] Zuiker R.G., Tribouley C., Diamant Z., Boot J.D., Cohen A.F., van Dyck K., De Lepeleire I., Rivas V.M., Malkov V.A., Burggraaf J. (2016). Eur. Clin. Respir. J..

[23] Yamauchi K. (1996). Nihon Rinsho..

[24] Kato K., Lillehoj E.P., Lu W., Kim K.C. (2017). J. Clin. Med..

[25] Damera G., Xia B., Sachdev G.P. (2006). Respiratory Res..

[26] Arathimos R., Granell R., Haycock P., Richmond R.C., Yarmolinsky J., Relton C.L., Tilling K. (2019). Thorax..

[27] Yen C.-F., Kim M.-R., Lee C.-L. (2019). Gynecol. Minim. Invasive Ther..

[28] Nwaru B.I., Tibble H., Shah S.A., Pillinger R., McLean S., Ryan D.P., Critchley H., Price D.B., Hawrylowicz C.M., Simpson C.R. (2021). Thorax..

[29] Chowdhury N.U., Guntur V.P., Newcomb D.C., Wechsler M.E. (2021). Eur. Respir. Rev..

[30] Yung J.A., Fuseini H., Newcomb D.C. (2018). Ann. Allergy Asthma Immunol..

[31] Amberger J.S., Bocchini C.A., Schiettecatte F., Scott A.F., Hamosh A. (2015). Nucleic Acids Research.

[32] Yoshikawa T., Nakamura T., Yanai K. (2019). Int. J. Mol. Sci..

[33] Szczepankiewicz A., Bręborowicz A., Sobkowiak P., Popiel A. (2010). Clin. Mol. Allergy..

[34] Freihat L.A., Wheeler J.I., Wong A., Turek I., Manallack D.T., Irving H.R. (2019). Sci. Rep. Nature..

[35] Matucci A., Bormioli S., Nencini F., Maggi E., Vultaggio A. (2021). Expert. Rev. Clin. Immunol..

[36] Borish L., Steinke J.W. (2011). Curr. Allergy Asthma Rep..

[37] Matera M.G., Rogliani P., Calzetta L., Cazzola M. (2020). Drugs..

[38] Gordon E.D., Palandra J., Wesolowska-Andersen A., Ringel L., Rios C.L., Lachowicz-Scroggins M.E., Sharp L.Z., Everman J.L., MacLeod H.J. (2016). JCI Insight..

[39] Zhang Y., Moffatt M.F., Cookson W.O.C. (2012). Curr. Opin. Pulmonary Med..

[40] Elena-Pérez S., Heredero-Jung D.H., García-Sánchez A., Estravís M., Martin M.J., Ramos-González J., Triviño J.C., Isidoro-García M., Sanz C., Dávila I. (2021). Front. Med. (Lausanne)..

[41] Ota K., Kawaguchi M., Matsukura S., Kurokawa M., Kokubu F., Fujita J., Morishima Y., Huang S.K., Ishii Y., Satoh H. (2014). J. Immunol. Res..

[42] Theron A.J., Steel H.C., Tintinger G.R., Gravett C.M., Anderson R., Feldman C. (2014). J. Immunol. Res..

[43] García-Solaesa V., Sanz-Lozano C., Padrón-Morales J., Hernández-Hernández L., García-Sánchez A., Rivera-Reigada M.L., Dávila-González I., Lorente-Toledano F., Isidoro-García M. (2014). Allergol. Immunopathol (Madrid)..

[44] (2022). Clin. Exp. Allergy..

[45] Nikolskii A.A., Shilovskiy I.P., Barvinskaia E.D., Korneev A.V., Sundukova M.S., Khaitov M.R. (2021). Biochemistry (Moscow)..

[46] Saik O.V., Demenkov P.S., Ivanisenko T.V., Bragina E.Y., Freidin M.B., Dosenko V.E., Zolotareva O.I., Choynzonov E.L., Hofestaedt R., Ivanisenko V.A. (2018). J. Integr. Bioinform..

[47] Trophimov V.I., Mineyev V.N., Sorokina L.N., Nema M.A., Lim V.V., Yeremeyeva A.V. Med. Acad. J. 2013. V. 13,.

[48] Pietras C.O., Vendelin J., Anedda F., Bruce S., Adner M., Sundman L., Pulkkinen V., Alenius H., D’Amato M., Söderhäll C. (2011). BMC Pulm. Med..

[49] Komi D.E.A., Kazemi T., Bussink A.P. (2016). Curr. Allergy Asthma Rep..

[50] Sleiman P.M., Flory J., Imielinski M., Bradfield J.P., Annaiah K., Willis-Owen S.A., Wang K., Rafaels N.M., Michel S., Bonnelykke K. (2010). N. Engl. J. Med..

[51] Fiuza B.S.D., de Silva M.J., Alcântara-Neves N.M., Barreto M.L., Costa R.D.S., Figueiredo C.A. (2017). Mol. Immunol..

[52] Sharma N., Tripathi P., Awasthi S. (2011). Allergy Rhinol. (Providence)..

[53] Paulenda T., Draber P. (2016). c.

[54] Luthers C.R., Dunn T.M., Snow A.L. (2020). Front. Immunol..

[55] Pavón-Romero G.F., Serrano-Pérez N.H., García-Sánchez L., Ramírez-Jiménez F., Terán L.M. (2021). Front. Cell. Dev. Biol..

[56] Ferraro V., Carraro S., Bozzetto S., Zanconato S., Baraldi E. (2018). Asthma Res. Pract..

[57] Batozhargalova B.T., Diakova S.E., Petrova N.V., Mizernitsky Yu.L., Zinchenko R.A. (2019). Russian Bulletin of Perinatology and Pediatrics..

[58] Bouzigon E., Monier F., Boussaha M., Le Moual N., Huyvaert H., Matran R., Letort S., Bousquet J., Pin I., Lathrop M. (2012). PLoS One..

[59] Sardaryan I.S., Zhelenina L.A., Galustyan A.N., Korostovtsev D.S., Ivashchenko T.E. (2008). Russian Bulletin of Perinatology and Pediatrics..

[60] Li X., Howard T.D., Zheng S.L., Haselkorn T., Peters S.P., Meyers D.A., Bleecker E.R. (2010). J. Allergy Clin. Immunol..

[61] White J.H., Chiano M., Wigglesworth M., Geske R., Riley J., White N., Hall S., Zhu G., Maurio F., Savage T. (2008). Human Molecular Genetics.

[62] Das S., Miller M., Beppu A.K., Mueller J., McGeough M.D., Vuong C., Karta M.R., Rosenthal P., Chouiali F., Doherty T.A. (2016). Proc. Natl. Acad. Sci. USA..

[63] Krautenbacher N., Flach N., Böck A., Laubhahn K., Laimighofer M., Theis F.J., Ankerst D.P., Fuchs C., Schaub B. (2019). Allergy..

[64] Wen X., Yan J., Han X.-R., Zheng G.-H., Tang R., Liu L.-F., Wu D.-M., Lu J., Zheng Y.-L. (2018). J. Thorac. Dis..

[65] Zhai W., Sun H., Li Z., Li L., Jin A., Li Y., Chen J., Yang X., Sun Q., Lu S. (2021). J. Immunol..

[66] Wancheng T., Weici L. (2001). Respirilogy..

[67] Yang M., Wu T., Cheng L., Wang F., Wei Q., Tanguay R.M. (2005). Respirat. Res..

[68] Hansel N.N., Diette G.B. (2007). Proc. Am. Thorac. Soc..

[69] Lee Y.-C., Jogie-Brahim S., Lee D.-Y., Han J., Harada A., Murphy L.J., Oh Y. (2011). J. Biol. Chem..

[70] Mcguckin M., Thornton D., Whitsett J. (2015). Mucosal Immunol. Cambridge: Acad. Press Books,.

[71] Bonser L.R., Erle D.J. (2017). J. Clin. Med..

[72] Zhang C., Yang P., Chen Y., Liu J., Yuan X. (2018). Exp. Ther. Med..

[73] Yildirim Yaroğlu H., Calikoğlu M., Tamer Gümüş L. (2011). Med. Princ. Pract..

[74] Niewiński P., Patkowski J., Orzechowska-Juzwenko K., Hurkacz M., Wolańczyk-Medrala A., Nittner-Marszalska M. (2005). Adv. Clin. Exp. Med..

[75] Yim E.-Y., Kang H.-R., Jung J.-W., Sohn S.-W., Cho S.-H. (2013). Asia PacAllergy..

[76] Ntontsi P., Photiades A., Zervas E., Xanthou G., Samitas K. (2021). Internat. J. Mol. Sci..

[77] Laitinen T. (2007). Meth. Mol. Biol..

[78] Liang S., Wei X., Gong C., Wei J., Chen Z., Chen X., Wang Z., Deng J. (2013). Respirology..

[79] Pawlik A., Juzyszyn Z., Gawronska-Szklarz B. (2009). Arch. Med. Res..

[80] Massa D., Baran M., Bengoechea J.A., Bowie A.G. (2020). J. Biol. Chem..

[81] DeVries A., Wlasiuk G., Miller S.J., Bosco A., Stern D.A., Lohman I.C., Rothers J., Jones A.C., Nicodemus-Johnson J., Vasquez M.M. (2017). J. Allergy Clin. Immunol..

[82] Stein M.M., Thompson E.E., Schoettler N., Helling B.A., Magnaye K.M., Stanhope C., Igartua C., Morin A., Washington C., Nicolae D. (2018). J. Allergy Clin. Immunol..

[83] Inoue H., Hattori T., Zhou X., Etling E.B., Modena B.D., Trudeau J.B., Holguin F., Wenzel S.E. (2019). J. Allergy Clin. Immunol..

[84] Maghsoudloo M., Azimzadeh Jamalkandi S., Najafi A., Masoudi-Nejad A. (2020). Mol. Med..

[85] Cardenas A., Sordillo J.E., Rifas-Shiman S.L., Chung W., Liang L., Coull B.A., Hivert M.-F., Lai P.S., Forno E., Celedón J.C. (2019). Nat. Commun..

[86] Li M., Tang Y., Zhao E.-Y., Chen C.-H., Dong L.-L. (2021). Zhongguo Dang Dai Er Ke Za Zhi..

[87] Hudon Thibeault A.-A., Laprise C. (2019). Genes (Basel)..

[88] Bai J., Zhong J.-Y., Liao W., Hu R., Chen L., Wu X.-J., Liu S.-P. (2020). Mol. Med. Rep..

[89] Gunawardhana L.P., Gibson P.G., Simpson J.L., Benton M.C., Lea R.A., Baines K.J. (2014). Epigenetics..

[90] Ray A., Cohn L. (1999). J. Clin. Invest..

[91] Vale-Pereira S., Todo-Bom A., Geraldes L., Schmidt-Weber C., Akdis C.A., Mota-Pinto A. (2011). Clin. Exp. Allergy..

[92] Hou C., Zhu X., Chang X. (2018). Exp. Ther. Med..

[93] Allen M., Heinzmann A., Noguchi E., Abecasis G., Broxholme J., Ponting C.P., Bhattacharyya S., Tinsley J., Zhang Y., Holt R. (2003). Nat. Genet..

[94] Holloway J.W., Beghe B., Holgate S.T. (1999). Clin. Exp. Allergy..

[95] Mukherjee M., Brown A., Pritchard D.I., Bosquillon C. (2013). Physiological Soc..

[96] Salam M.T., Lin P.-C., Avol E.L., Gauderman W.J., Gilliland F.D. (2007). Thorax..

[97] Zheng Y., Wang H., Luo L., Liao L., You L., Wang J., Li Q. (2018). Medicine (Baltimore)..

[98] Ohbayashi H., Shimokata K. (2005). Curr. Drug Targets Inflamm. Allergy..

[99] Averyanov A.B., Cherkashina I.I., Nikulina S.Yu., Maksimov V.N., Shestovitsky V.A. (2019). Therapeutic archive..

[100] Chan M.A., Gigliotti N.M., Aubin B.G., Rosenwasser L.J. (2014). Am. J. Respir. Cell. Mol. Biol..

[101] Ding Q., Sun S., Zhang Y., Tang P., Lv C., Ma H., Yu Y., Xu S., Deng Z. (2020). COPD..

[102] Israel E., Lasky-Su J., Markezich A., Damask A., Szefler S.J., Schuemann B., Klanderman B., Sylvia J., Kazani S., Wu R. (2015). Am. J. Respir. Crit. Care Med..

[103] Kachroo P., Hecker J., Chawes B.L., Ahluwalia T.S., Cho M.H., Qiao D., Kelly R.S., Chu S.H., Virkud Y.V., Huang M. (2019). Nat. Heart, Lung, Blood Inst. Trans- Omics for Precision Med. Consortium..

[104] Hernandez-Pacheco N., Farzan N., Francis B., Karimi L., Repnik K., Vijverberg S.J., Soares P., Schieck M., Gorenjak M., Forno E. (2019). Clin. Exp. Allergy..

[105] Perez-Garcia J., Espuela-Ortiz A., Lorenzo-Diaz F., Pino-Yanes M. (2020). Pharmgenomics Pers. Med..

[106] Lee J.H., McDonald M.-L.N., Cho M.H., Wan E.S., Castaldi P.J., Hunninghake G.M., Marchetti N., Lynch D.A., Crapo J.D., Lomas D.A. (2014). Respir. Res..

[107] Dengler H.S., Wu X., Peng I., Rinderknecht C.H., Kwon Y., Suto E., Kohli P.B., Liimatta M., Barrett K., Lloyd J. (2018). Sci. Transl. Med..

[108] Tabèze L., Marchand-Adam S., Borie R., Justet A., Dupin C., Dombret M.-C., Crestani B., Taillé C. (2019). Eur. Respir. J..

[109] Finsterer J. (2019). Orphanet. J. Rare Dis..

[110] Al-Alawi M., Hassan T., Chotirmall S.H. (2014). Respir. Med..

[111] Li X., Howard T.D., Moore W.C., Ampleford E.J., Li H., Busse W.W., Calhoun W.J., Castro M., Chung K.F., Erzurum S.C. (2011). J. Allergy Clin. Immunol..

[112] Gaurav R., Varasteh J.T., Weaver M.R., Jacobson S.R., Hernandez-Lagunas L., Liu Q., Nozik-Grayck E., Chu H.W., Alam R., Nordestgaard B.G. (2017). JCI Insight..

[113] Smit J.J., Folkerts G., Nijkamp F.P. (2004). Trends Immunol..

[114] Moussette S., Al Tuwaijri A., Kohan-Ghadr H.-R., Elzein S., Farias R., Bérubé J., Ho B., Laprise C., Goodyer C.G., Rousseau S. (2017). PLoS One..

